# Fairness and Smiling Mediate the Effects of Openness on Perceived Fairness: Beside Perceived Intention

**DOI:** 10.3389/fpsyg.2018.00772

**Published:** 2018-05-23

**Authors:** Zhifang He, Jianping Liu, Zhiming Rao, Lili Wan

**Affiliations:** ^1^School of Psychology, Jiangxi Normal University, Nanchang, China; ^2^School of Humanities, Jiangxi University of Traditional Chinese Medicine, Nanchang, China; ^3^School of Physics and Communication Electronics, Jiangxi Normal University, Nanchang, China

**Keywords:** smiling, openness, perceived intention, fairness, ultimatum game

## Abstract

Previous studies have shown that smiling, fairness, intention, and the results being openness to the proposer can influence the responses in ultimatum games, respectively. But it is not clear that how the four factors might interact with each other in twos or in threes or in fours. This study examined the way that how the four factors work in resource distribution games by testing the differences between average rejection rates in different treatments. Two hundred and twenty healthy volunteers participated in an intentional version of the ultimatum game (UG). The experiment used a 2 × 2 × 2 × 2 mixed design with “openness” as a between subjects factor and the other three as within subjects factors, and the participants were assigned as recipients. The results revealed that fairness or perceived good intention reduced the subject’s average rejection rates. There was a significant interaction between facial expressions and openness. With fair offers, the average rejection rate for informed was lower than that of uninformed; but when unfair, no difference between the corresponding average rejection rates was found. The interaction effect of smiling and openness was also significant, the average rejection rate for informed offers was lower when the proposer was smiling and no rejection rate difference between uninformed offers and informed offers when no smiling. No other interaction effect was found.

## Introduction

Fairness is of utmost importance in social life, as well as in political and economic life (Carl et al., 2006). Defined as the phenomenon of inequity aversion, violation of the social norm of fairness can elicit negative emotions (Stouten et al., 2011) and give rise to subsequent strong reactions, including punishment (Mendoza et al., 2014) or even personal revenge for unequal distributions of resources. Different theories were developed to explain why some people feel more fairness than others as they are facing the same distribution. Utility theory was first proposed with the rationality hypothesis, suggesting that when faced with resource distribution, people tend to make choices with greater utility (Fishburn, 1967). Later, implicit expected utility theory was proposed with an implicit economic cognition hypothesis, which takes effects into account in the decision process model (Raaij and Ye, 2002). However, utility is not the only thing that people consider when making decisions. Studies on belief in a just world (Lerner, 1965), defensive attributions (Shaver, 1970), retributive justice (Darley and Pittman, 2003), criminal responsibility (Gebotys and Dasgupta, 1987), and moral psychology (Gray and Wegner, 2010; Haidt and Kesebir, 2010; Knobe et al., 2012) all converge to show that when people detect harm, they become motivated to blame someone for that harm. It has been found that a receiver’s perception of the intention of a distributor affects the receiver’s sense of fairness (Güroglu et al., 2011) and that perceived good intention alleviates the sense of unfairness (Ma et al., 2015b). Numerous behavioral and neuroscientific experiments have demonstrated that intentional harms make people want to blame, condemn, and punish more than unintentional harms do (Alicke, 1992; Darley and Pittman, 2003; Young and Saxe, 2009). People are notoriously sensitive to harmful intentions (Gollwitzer et al., 2009), and even exposure to fictional characters with harmful intentions can change subsequent trust behavior in real life (Rothmund et al., 2011). Intention plays an important role and might lead to sequential reciprocity (Dufwenberg and Kirchsteiger, 1998). However, because people cannot observe others’ intention, intention is only perceived. Here in this paper, we use the term “perceived intention." The conclusions of perceived intention are diverse. One study showed that perceived intention was consistent with the reciprocity hypothesis (McCabe et al., 2003), which overthrew the previous conclusion that perceived intention was closely related to the experimental results, that is, the sum of money gained by the subjects. Another experiment showed that certain outcomes, along with intentions and motivations, account for reciprocity (Stanca et al., 2009).

Facial expressions are informative and expressive in social interactions, and they help the receivers reason, judge and make decisions during social interactions, and have a function in social interaction. Smiling expressions were found to reduce the perceived anger (Bugental, 1974), and different smiling models might lead to different reactions (Krumhuber et al., 2009). Smiling offers were more likely to be accepted (Mussel et al., 2013). As for fairness, facial expressions impact the decision making concerning fairness (Mussel et al., 2014). In real life, the emotional state of a distributor may affect the allocation of resources, and the perceived emotions of a distributor will also have an impact on the fairness perceived by the recipient. In face-to-face communication, the recognition of facial expressions is an important way to judge the emotion of the two sides, and it is also an important social cue that affects the psychological process of the communicator. A smiling expression might facilitate trust (van’t Wout and Sanfey, 2008) and lead to cooperation. One’s emotion may play a part in perceived fairness (Heussler et al., 2009); therefore, the reason that why one’s partner’s emotion might influence one’s own emotion and thus affects perceived fairness seems logical.

Fairness evolving during resource distribution is linked to reputation, which concerns proposer’s knowledge of responder’s deal (Nowak et al., 2000). When it comes to social affairs, or public goods, information is of the most importance. If the proposers will be notified of what responders have done and the responders know it, an education will happen to teach the proposers a lesson (Abbink et al., 2004), and in time fairness will finally be done. Though people could deduce others’ intention and emotion from their expression, they can’t predict the corresponding behaviors. So during UG time, if proposers know these behaviors, the following distributions may be different. And if the responders know what they have done will get to the proposers, they may act another way. But whether or how the effect of openness will be affected by perceived intention, fairness, and expression on perceived fairness remains unknown. We regard that openness may urge the responders to show their moral courage and to make decisions more for public goods, and Chinese traditional culture such as “be wordly wise and play safely” may also take its place. The study of openness in perceived fairness is relatively fewer compared with intention, fairness or emotion. Whether the openness in resource distribution would be counterbalanced by the Chinese traditional culture remains unknown, so our principle concern in this paper is openness.

We also wonder that if openness meets obviously unfair in resource distribution, what would happen? And still, what it would be if openness meets a smiling face? Does the Chinese saying “Don’t be angry to the person in smiles” still works in a resource distribution experiment? And as “Don’t lose face” has extraordinary personal meaning and “Do boldly what is righteous” is of important social meaning in China, we wonder if an unfair offer together with openness and a smiling face would affect the responders’ decisions.

The ultimatum game (UG) (Güth et al., 1982), measures decision-making in a resource distribution context. A classic UG has two roles, a proposer, a responder, and a certain amount of stake. The proposer receives the stake and has to make an arbitrary offer to share with the responder. The responder decides to accept or to reject the offer. If the offer is accepted, both of them receive payment as the offer requires, if it is rejected, both receive nothing. For example, a proposer divides $10 among himself and a responder, then the responder decides whether to reject the proposal so that neither player receives anything, or to accept the offer, so that each player gets her/his money according to the division. During the UG, the proposer decides the distribution of the stake, and the responder decides whether the offer works. UG concerns about resource distribution, social comparison and people’s decision making, so we can say that the experimental paradigm is logically suit for the purpose of perceived fairness study.

Widely used to examine people’s responses to unfairness, the UG is often modified for the purpose of different experiments. In this study, the variation in the ultimatum game was used to investigate the effects, especially the interact effects of fairness, perceived intention, smiling and the openness of a responder’s responses on perceived fairness. For each participant, a certain amount of money was divided between a proposer and a responder (Güth et al., 1983). We made our experiment different from the common paradigm of the ultimatum game in that each time, two possible divisions were present. The proposer decided how to divide the money, and the responder decided whether to accept or reject the offer. We aimed to test whether the effect of the openness of the responder’s decision on perceived fairness was moderated by the facial expression of the distributor (the proposer) and/or the fairness of the distribution. Perceived fairness was measured by participants’ rejection rates of the distribution. The hypotheses are: (1) Fairness promotes the perceived fairness. This would be manifested by the lower rejection rate for fairness. (2) Perceived good intention reduces the perceived fairness. This would supported by a corresponding lower rejection rate for perceived good intention. (3) Fairness moderates the effect of openness. Evidence would come from that the difference between the rejection rates for fair informed vs. fair uninformed distributions is different from that of the rejection rates for unfair informed vs. unfair uninformed distributions. (4) Smiling moderates the effect of openness, it will be proved by that the difference between the rejection rates for smiling informed vs. smiling uninformed distributions is different from that of the rejection rates for no-smiling informed vs. no-smiling uninformed distributions.

## Materials and Methods

### Participants

To get adequate power of statistics (above 0.8), we used G^∗^Power 3 software (Blue et al., 2016) and it suggested a size of no less of 199 for this study to get a medium-size effect (*f* = 0.20). 260 healthy volunteers (undergraduates) were recruited from two universities in Nanchang, none of whom were from psychology or social disciplines. We made it clear during the recruiting that only those who had never taken part in experiments involving UG were qualified. We excluded 40 participants’ data after the UG experiment because they failed the trust check for their disbelief in the truth of the experiment. Thus, the final sample included 220 students (109 females) aged 18–25 (mean age = 21.5, SD = 1.6). The experiment was conducted in accordance with the Declaration of Xiaoman Yan and was approved by the Ethics Committee of Jiangxi University of Traditional Chinese Medicine. We collected informed written consent from every participant prior to the experiment.

## Materials

### Experimental Design and Procedure

Participants were divided into groups of 10–15 persons. On arrival, participants were told that they would play a money distribution game with partners online. They were also told that all players would be anonymous and that a blurry facial expression image would be assigned to the player. Each time, an assistant guided a group of participants to the psychological laboratory, and they were notified that they were specified randomly as the recipients. Every subject was seated in front of a screen, which was 100 cm in front of him. The stimulus was presented at the center of the screen, and the visual angle was about 8° × 7°. Half of the subjects were instructed to use “J” for “agree” and “F” for “reject,” and the rest were the versus. When one finished her/his task, reward would be paid.

### Design

The experiment had a 2 × 2 × 2 × 2 mixed design. The first factor, facial expression, had two levels, smiling vs. no-smiling, which was conveyed by a facial image on the screen. The number of images was balanced in terms of the sex and emotion of the proposers. No image was repeated during one participant’s experiment. The second factor was the fairness of the distribution, fair vs. unfair, which was determined by the distribution rate. For example, the rate could be 6:4 (the proposer took six yuan out of 10 yuan), a relatively fair distribution, or 8:2, a rather unfair one. Other rates are shown in table one. The third factor was the proposer’s perceived intention, good intention vs. bad intention, which was conveyed through the proposer’s choice. The proposer made a choice between two rates, and if the proposer chose the option to maximize his/her own profit, the subject sensed bad intentions. For example, for 5:5 vs. 6:4, if the proposer chose 6:4 (thus receiving 6 out of 10 yuan), the recipient perceived bad intentions because the proposer did not choose a less selfish distribution. If the proposer chose 5:5, then the proposer received less and the recipient received more than if the proposer chose 6:4. Thus, the recipient perceived good intentions. The fourth, the only between factor, was the openness of the responder’s decision, informed vs. uniformed. The subjects were randomly assigned to an informed group or an uninformed group. For more details on the stimulus design, see **Table 1**.

**Table 1:** The stimulus design.

Facial expression	Fairness:Paired rates	perceived intention	trials
Smiling	5:5 vs. 6:4 fair	Bad	20
Smiling	6:4 vs.7:3 fair	Good	20
Smiling	8:2 vs. 9:1 unfair	Bad	20
Smiling	9:1 vs 10:0 unfair	Good	20
No-smiling	5:5 vs. 6:4 fair	Bad	20
No-smiling	6:4 vs.7:3 fair	Good	20
No-smiling	8:2 vs. 9:1 unfair	Bad	20
No-smiling	9:1 vs. 10:0 unfair	Good	20

*If chosen, the underlined numbers are targets, which produce the data for analysis, and the corresponding ones, if chosen, are masks. In x:y, x is the money given to the provider, and y is the subject.*

The present experiment was a modified mini UG paradigm (Falk et al., 2003). We made it different from the mini UG that different unfair distributions were present, and the unfair alternative rates of 9:1 vs. 10:0 were more extreme. The computer presented the distribution rates randomly. Each distribution was a pair of rates in **Table 1** and was presented the same number of times. In each pair, the rates were chosen an equal number of times. Therefore, the target rates were presented twice for the corresponding masking ones. The whole experiment consisted of 16 blocks, and each block included 10 trials. One hundred sixty emotional images (80 of them are smiling, half of them are female) were used, and no faces were of the same person. As the proposer’s facial attractiveness matters during the UG (Ma et al., 2015a), we balanced the attractiveness by a procedure that let the attractiveness assessed on LAN scoring from 0 to 10 before the experiment. The assessors were freshmen and no one would join the experiment. Each picture was scored by a group of assessors, ten males and ten females. Each group assessed twenty pictures (the number of smiling females was 5, for the sake of balance). We hand-picked 160 pictures scored between 5 to 8 out of 500 pictures. AOV of the scores showed no difference, *F*(3,159) = 1.745, *p* > 0.05.

## Procedure

**FIGURE 1:**
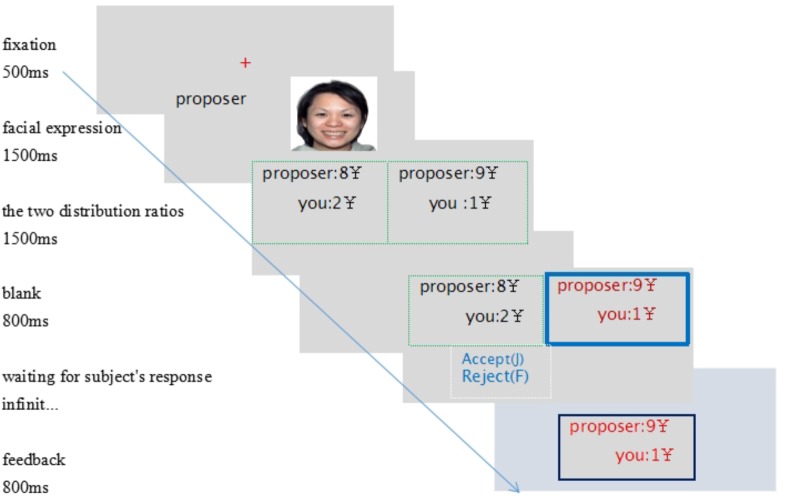
Experimental task. (1) Fixation; (2) the proposer’s facial expression; (3) the alternative division; (4) the proposer’s thinking; (5) the subject’s decision; (6) feedback.

The participants were told the rules of the UG. As shown in **Figure 1**, the fixation point (a red “+”) shown for 500 ms at the center of the black screen indicated that the stimulus would soon be presented. Then, the proposer’s facial image was displayed on the screen for 1500 ms. Next, the distribution was shown on the screen for 1500 ms. A blank screen was shown for 800 ms, meaning that the proposer was thinking, and the distribution showed up again, with the numbers colored in the bold frame as the proposer’s choice. The participant pressed “F” to reject or press “J” to accept the offer. If the distribution was rejected, both the proposer and the receipt received nothing, and if it was accepted, each received the money, distributed as the proposer decided. The feedback was on the screen for 800 ms. Every participant performed four practice trials to become familiar with the experimental procedure before the formal experiment began. When the experiment was over, each participant completed a form to check whether he/she believed it was a real bargain. Each participant received 30 yuan (about 4.4 USD) for attendance, and extra decision-based payment was decided by two randomly selected of the participant’s trials. On leaving, the amount was calculated and the participant was paid on the spot. The whole process was programmed with E-prime 2.0 software.

## Results

**FIGURE 2:**
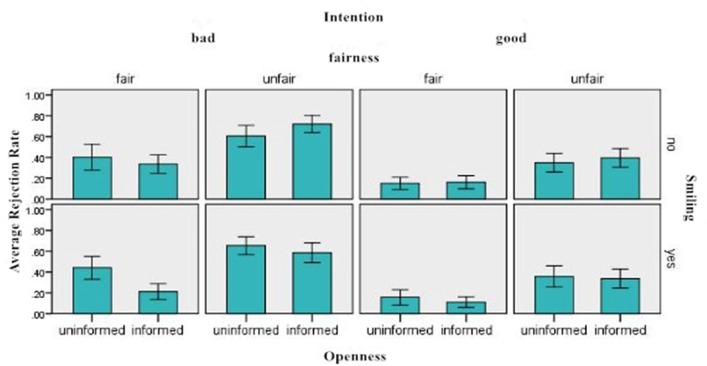
The average rejection rates as a function of intention, fairness, expression, and openness.

The rejection rates under different conditions are shown in **Figure 2**.

We performed a 2 (facial expression:smiling vs. non-smiling) × 2 (fairness:fair vs. unfair) × 2 (openness:informed vs. uninformed) × 2 (perceived intention:good vs. bad) repeated ANOVA on subjects’ rejection rates for different offers in UG. The analysis revealed a significant main effect of fairness, *F*(1,218) = 118.771, *p* < 0.001, partial η^2^ = 0.144, with the rejection rate for fair offers (0.2470 ± 0.0162, CI = [0.2788, 0.2152]) lower than the one for unfair offers (0.5011 ± 0.0159,CI = [0.5323, 0.4699]). The main effect of perceived intention was also significant, *F*(1,218) = 107.846, *p* < 0.001, partial η^2^ = 0.133, with the rejection rate was lower for perceived good intention (0.2532 ± 0.0160, CI = [0.2846, 0.2218]) than for unfair offers(0.4953 ± 0.0160, CI = [0.5267, 0.4639]). No significant main effect of other factors was found. There was a significant interaction between fairness and openness, *F*(1,218) = 4.663, *p* < 0.05, partial η^2^ = 0.007. Simple effects tests showed that if the offers were fair, the rejection rate for uninformed offers(0.2886 ± 0.0269, CI = [0.3413, 0.2359]) was significant higher than that of informed ones(0.2056 ± 0.0190, CI = [0.1682, 0.2430]), *p* < 0.05, *F*(1,218) = 6.335, partial η^2^ = 0.009); when the offers were unfair, the corresponding average rejection rates were not significantly different, *p* = 0.5922. The interaction effect of smiling and openness on average rejection rate was also significant, *F*(1,218) = 6.396, *p* < 0.05, partial η^2^ = 0.009. The proceeding simple effects test showed the average rejection rate for uninformed offers was higher (0.4031 ± 0.0269, CI = [0.3502, 0.4559]) than that for informed ones(0.3115 ± 0.0190, CI = [0.2741, 0.3489]) when the proposer was smiling, *p* < 0.01, partial η^2^= 0.011; there was no difference between the average rejection rate of uninformed offers and that of informed offers when the proposer didn’t smile, *p* = 0.5436. No other two way interaction effect was found, and no any three way effect or four way effect was found either.

## Discussion

The data showed that fairness and perceived intention had significant effects on perceived fairness. So Hypothesis I and Hypothesis II were proved. In the distribution of resources, a fairer distribution led to a lower average rejection rate, which can be explained by utility theory (French, 2006) or unfair aversion model (Fehr and Schmidt, 1999). Unfair monetary UG offers elicit anger and might result in rejection (Gilam et al., 2015). Utility theory assumes the preferences of utility when people decide among alternatives. In our experiment, fairness meant more favorable outcomes (or more utility) for the receiver, so it is natural that fairness led to a low average rejection rate.

Perceived good intention tends to increase perceived fairness. Researchers have shown that procedural fairness has a considerable influence on employees’ attitudes toward their organization and its members (Brockner et al., 2003). We deduced that perceived intention in our experiment might partly refer to procedural unfairness, which was uncontrollable for the receiver but controllable for the proposer. Perceived bad intention also induced angry and retaliatory behavior, so when the proposer made an unfair decision, the bad intention perceived by the receiver may have resulted in a relatively high average rejection rate. Or, as someone puts it (Rabin, 1993): Fairness means that if you are kind to me, I will be kind to you, but if you mean bad to me, then I will do the same to you. So the concept of perceived intention directly penetrates the meaning of fairness.

Interaction between fairness and openness was significant, as the data showed, with the average rejection rate for fair, uninformed offers higher than fair, informed ones, Hypothesis III was manifested. This may because the fair distributions are “should be taken ones” and reject them may be viewed as either wicked or unwise, so more offers were rejected if anonymous. To some extent, it may also be attributed to Chinese culture: Chinese people refuse relatively less in public. The mentality of ‘Don’t lose face’ or ‘worldly wise’ was severe in China, so informed fair offers were accepted more easily: accepting the offers under the openness condition meant saving the proposer’s face, that would finally help the responder himself/herself. As for fair and uninformed offers, it was always safe, so the responders might feel freer to act as what they are pleased. We reasoned that when unfair distribution appeared, an anchoring effect (Strack and Mussweiler, 1997) might occur and the informed or uninformed offers were indistinguishably treated. That is, unfairness was the most important working information for judgement. This might mean other factors had little effect when unfair distribution occurred, the final decision tended to favor the effect of unfairness. One might anticipate logically that when it’s unfair, the spirit of “Do boldly what is righteous for public good” should work and lead to more average rejections of informed offers, as other researchers had described (Abbink et al., 2004). But this didn’t happen. However, it didn’t mean that more financial considerations than moral ones prevailed in the decision making. The unfair offers did take the form of an anchoring effect, but “safely play” counteracted the moral concern under informed condition was another possible additional reason. This might explain why our responders didn’t teach a lesson more often when unfair, informed offers provided than when unfair, uninformed ones.

The interaction effect of smiling and openness was also significant, with the smiling average rejection rate for uninformed offers was higher than that for informed ones. Hypothesis IV was manifested. According to the spreading-activation theory (Loftus, 1975), the awakening of a semantic concept will activate related concepts in the neural network simultaneously. Fairness perception relates to emotions (Namkung and Jang, 2010), upon observing a smiling face, the anchoring effect bias (Bennett, 2014) took place, concepts such as “good person,” “pleasure to see” and “like” might be activated. We reasoned that smiling stirred good feelings, and when the acceptances were open, and the reponders were more likely to convey a kind repay. When the smiling expression appeared the responder might take it as the intrinsic nature of the proposer (Chee and Murachver, 2012), and if anonymous was available, to teach a lesson was a natural and safe action, and also, a noble decision. This anchoring effect was different from the traditional Chinese culture of “Don’t be angry to the person in smiles,” which means people tend to forgive those who apologize honestly. Our outcome might partly attribute to the traditional Chinese culture: when the decision would be sent to the proposer, declining an offer from a smiling face would easily get into an embarrassed situation that most Chinese people would try to avoid. So the corresponding average rejection rate was lower than that of smiling but uniformed offers. The anchoring effect of smiling was thus revised. According to attribution theory (Kelley, 1967), a no-smiling expression might show that the proposer does not have control, so openness didn’t make difference. It was like in price-fairness experiments, price increases were perceived as less fair when the causality was directly attributable to the seller’s controllable actions (Vaidyanathan and Aggarwal, 2003). Chinese people are easy to forgive, especially when the wrongdoers are forced to do, this we attributed to a strong Chinese traditional culture of “forgive wherever you can.” That was a possible reason for equally rejected no-smiling, informed.

## Conclusion

We found the fairness of a distribution itself affects perceived fairness. The fairer the distribution, the lower the average rejection rate. The distributor’s perceived good intention leads to a lower average rejection rate, as the results show. We also found that smiling facial expressions moderate the effect of openness: smiling and openness lead to a lower average rejection rate, and fairness moderate the effect of openness: it is beneficial for the proposer to smile when he/she could get the information of the responder’s decision for the sake of the offer to be accepted.

## Author Contributions

ZH and JL designed the experiments. ZH and ZR collected the data. ZH, JL, ZR, and LW wrote the manuscript.

## Conflict of Interest Statement

The authors declare that the research was conducted in the absence of any commercial or financial relationships that could be construed as a potential conflict of interest. The reviewer XL and handling Editor declared their shared affiliation.
